# *Plasmodium falciparum *resistance to anti-malarial drugs in Papua New Guinea: evaluation of a community-based approach for the molecular monitoring of resistance

**DOI:** 10.1186/1475-2875-9-8

**Published:** 2010-01-07

**Authors:** Jutta Marfurt, Thomas A Smith, Ian M Hastings, Ivo Müller, Albert Sie, Olive Oa, Moses Baisor, John C Reeder, Hans-Peter Beck, Blaise Genton

**Affiliations:** 1 Swiss Tropical Institute, Department of Medical Parasitology and Infection Biology, and Department of Public Health and Epidemiology, Socinstrasse 57, PO Box, CH-4002 Basel, Switzerland; 2 Liverpool School of Tropical Medicine, Pembroke Place, Liverpool L3 5QA, UK; 3 Papua New Guinea Institute of Medical Research, Goroka, PO Box 60, EHP 441, Papua New Guinea

## Abstract

**Background:**

Molecular monitoring of parasite resistance has become an important complementary tool in establishing rational anti-malarial drug policies. Community surveys provide a representative sample of the parasite population and can be carried out more rapidly than accrual of samples from clinical cases, but it is not known whether the frequencies of genetic resistance markers in clinical cases differ from those in the overall population, or whether such community surveys can provide good predictions of treatment failure rates.

**Methods:**

Between 2003 and 2005, *in vivo *drug efficacy of amodiaquine or chloroquine plus sulphadoxine-pyrimethamine was determined at three sites in Papua New Guinea. The genetic drug resistance profile (i.e., 33 single nucleotide polymorphisms in *Plasmodium falciparum crt*, *mdr1*, *dhfr*, *dhps*, and *ATPase6*) was concurrently assessed in 639 community samples collected in the catchment areas of the respective health facilities by using a DNA microarray-based method. Mutant allele and haplotype frequencies were determined and their relationship with treatment failure rates at each site in each year was investigated.

**Results:**

PCR-corrected *in vivo *treatment failure rates were between 12% and 28% and varied by site and year with variable longitudinal trends. In the community samples, the frequencies of mutations in *pfcrt *and *pfmdr1 *were high and did not show significant changes over time. Mutant allele frequencies in *pfdhfr *were moderate and those in *pfdhps *were low. No mutations were detected in *pfATPase6*. There was much more variation between sites than temporal, within-site, variation in allele and haplotype frequencies. This variation did not correlate well with treatment failure rates. Allele and haplotype frequencies were very similar in clinical and community samples from the same site.

**Conclusions:**

The relationship between parasite genetics and *in vivo *treatment failure rate is not straightforward. The frequencies of genetic anti-malarial resistance markers appear to be very similar in community and clinical samples, but cannot be used to make precise predictions of clinical outcome. Thus, indicators based on molecular data have to be considered with caution and interpreted in the local context, especially with regard to prior drug usage and level of pre-existing immunity. Testing community samples for molecular drug resistance markers is a complementary tool that should help decision-making for the best treatment options and appropriate potential alternatives.

## Background

The development and spread of *Plasmodium falciparum *resistance to the most commonly used anti-malarial drugs is a major challenge in the control of malaria [1]. Quantifying the level of drug resistance through regular monitoring provides essential information to the health authorities responsible for ensuring ready access to effective drugs. Methods for assessing drug resistance in malaria include *in vivo *drug efficacy studies (the gold standard), *in vitro *sensitivity testing of patient isolates, and surveys designed to detect molecular markers of drug resistance. Each approach has advantages and disadvantages (discussed in [2-4]) and herein field data evaluating the usefulness of the molecular marker approach are presented.

Molecular screening to detect drug resistance was made possible by studies identifying the genetic basis of drug resistance in *P. falciparum *and this approach has subsequently become an integral part for the evaluation of resistance to treatment [5,6]. Chloroquine resistance (CQR) is attributable to single nucleotide polymorphisms (SNPs) in *pfcrt *and *pfmdr1 *(reviewed in [7-9]). Resistance to sulphadoxine-pyrimethamine (SP) is associated with a stepwise accumulation of mutations in *pfdhfr *and *pfdhps *(reviewed in [10]). Artemisinin derivates have been shown to inhibit *pfATPase6 *[11] and SNPs in *pfATPase6 *may become associated with resistance *in vitro *[12,13]. It has also become apparent that these mutations do not act in isolation, but often act synergistically to encode or enhance resistance. Resistance may arise through sequential accumulation of mutations in a single gene, such as anti-folate resistance in the *pfdhfr *gene [4,14]. Mutations in different genes may also act synergistically. Linkage disequilibrium is often observed between *pfcrt*K76T and *pfmdr1*N86Y *in vivo *[15-17] and the commonly accepted hypothesis is that CQR is mediated by multigenic processes [18], primary resistance being encoded by *pfcrt*K76T with SNPs in *pfmdr1 *playing an important role in modulating levels of CQR. The requirement to consider intra- and inter-genic genotypes impose limitations on the use of molecular methods such as PCR-RFLP which can identify the presence of mutations at individual markers, but not individual malaria genotypes defined at multiple loci in large sample sets.

The link between molecular markers and resistance can be clearly demonstrated in the laboratory, but the usefulness of these markers in the field has been controversial, primarily because the strength of their associations with *in vivo *treatment outcome is not consistent in different epidemiological settings. There are two plausible reasons for this [3]. Firstly, drug failure may occur for reasons other than parasite genetics. Patients may take incomplete drug courses, may have poor drug absorption or metabolism and so on. When marker frequencies are low, these human sources of drug failure may obscure the relationship between marker and resistance. Secondly, most studies have primarily focused on single genes and markers [19], rather than multiple markers which may act synergistically. A further limitation is that most analyses of molecular markers have been conducted as part of clinical studies and have, therefore, been limited to sentinel sites with good access to health care facilities and mostly examined clinical cases within a restricted age group [20]. Moreover, most such studies used parasite genetic measures based on the proportions of patients carrying a given genetic marker, or composite measures formed from *ad hoc *functions of such proportions [5]. This ignores the fact that patients often carry multiple clones of parasites and so may carry a mutation even if most of their parasites are wild-type. In principle, the evolution of drug resistance should be assessed by genetic profiling of representative samples of the circulating parasite population, which includes the reservoir in asymptomatic carriers. This should be summarized using population-genetic measures of allele frequencies (for single alleles) and haplotype frequencies (for multiple linked alleles). However, genetic data from community samples have rarely been compared with *in vivo *treatment responses [21,22]. In addition, it is not clear whether the molecular profile of parasites in symptomatic patients seen at health facilities matches that of the large circulating parasite reservoir.

The current study has investigated the role and applicability of the molecular drug resistance profiles in community samples for the monitoring of drug resistant malaria in Papua New Guinea (PNG). For this purpose, *in vivo *drug efficacy studies with the first-line regimen of amodiaquine or chloroquine plus sulphadoxine-pyrimethamine (AQ+SP or CQ+SP) were conducted at three different sites. A DNA microarray-based technology was used to compare the molecular drug resistance profile in clinical samples and blood samples collected in the communities from the catchment areas of the corresponding health facilities. Finally, the relationships of the allele and haplotype frequencies of the molecular markers with the rates and time-trends in clinical failure at the different sites were investigated.

## Methods

### Study areas and design

The three study sites in Papua New Guinea (PNG) were 1) the Sigimaru health centre (HC) in the Karimui area (Simbu Province), 2) the Kunjingini HC in the South Wosera area (East Sepik Province), and 3) the Mugil HC in the North Coast area of Madang (Madang Province). In Karimui, a rural region in the highland fringe area of PNG, the studies were run between October and April in three consecutive years (2003, 2004, and 2005). In the Wosera, located in the floodplain of the Sepik river in the north-eastern part of the country bordering Indonesian Papua, the study period was between December and June in 2003 and 2004. The study in the rainforest area at the North Coast of Madang was conducted between April 2004 and February 2005.

Malaria transmission is perennial with limited variations between wet (October to April) and dry (May to September) season at all sites. Transmission intensity in PNG decreases significantly with increasing altitude [23] and is higher in the lowland regions of the Wosera and the North Coast than in the Karimui area, an elevated plateau situated at an altitude of 700 to 1,200 m. There is little socioeconomic stratification between and within sites, although there are differences in health care provision and drug use patterns [24-28]. Baseline characteristics of the study sites are summarized in Table 1.

**Table 1:** Characteristics of study sites and populations between 2003 and 2005

	Karimui area (Simbu Province)			South Wosera (East Sepik Province)		North Coast (Madang Province)
Endemicity*	mesoendemic			mesoendemic		Mesoendemic
**Transmission intensity^§^**	moderate			high		high
**Year**	2003	2004	2005	2003	2004	2004
**n**	265	347	359	317	366	359
**Mean age in years** **(95% CI, range)**	18.9 (17.0-20.7, 0.5-60)	19.4 (17.7-21.2, 0.5-73)	15.4 (14.0-16.8, 0.5-66)	20.4 (18.7-22.2, 0.5-70)	21.3 (19.6-23.1, 0.5-69)	19.6 (17.8-21.5, 0.5-70)
**Sex: females/n (%)**	137/265 (51.7)	192/347 (55.3)	186/359 (51.9)	159/317 (50.1)	189/366 (51.6)	182/359 (50.7)
**Mean temperature in°C (95% CI)**	36.1 (36.0-36.1)	36.1 (36.0-36.1)	36.4 (36.4-36.5)	36.6 (36.5-36.6)	36.5 (36.5-36.6)	36.4 (36.3-36.4)
**Mean Hb in g/dl (95% CI)**	11.5 (11.2-11.8)	12.1 (11.8-12.3)	11.4 (11.2-11.7)	10.7 (10.5-10.9)	10.7 (10.6-10.9)	10.4 (10.3-10.6)
**x/n (Pf prevalence by microscopy** **(%, 95% CI)**	34/258 (13.2, 9.3-17.9)	64/346 (18.5, 14.5-23.0)	82/358 (22.9, 18.7-27.6)	55/314 (17.5, 13.5-22.2)	96/356 (27.0, 22.4-31.9)	82/358 (22.9, 18.7-27.6)
**x/n (Pf prevalence by *msp2 *nPCR** **(%, 95% CI)**	102/263 (38.8, 32.9-45.0)	71/347 (20.5, 16.3-25.1)	131/359 (36.5, 31.5-41.7)	129/317 (40.7, 35.2-46.3)	147/366 (40.2, 35.1-45.4)	115/359 (32.0, 27.2-37.1)
**Mean multiplicity of infection (MOI) (95% CI, range)**	1.46 (1.32-1.60, 1-4)	1.59 (1.38-1.80, 1-4)	1.77 (1.62-1.92, 1-4)	1.77 (1.57-1.96, 1-6)	1.85 (1.68-2.02, 1-5)	1.54 (1.38-1.70, 1-5)
* x/n (Pf prevalence in age group 2-9 (%, 95% CI)	13/93 (14.0, 7.7-22.8)	20/107 (18.7, 11.8-27.4)	44/138 (31.9, 24.2-40.4)	21/98 (23.6, 15.2-33.8)	36/110 (32.7, 24.1-42.3)	46/128 (35.9, 27.7-44.9)
* Spleen rate in age group 2-9 **(95% CI)**	nd	26.8 (18.8-36.2)	35.8 (27.7-44.6)	17.7 (10.2-27.4)	17.3 (10.7-25.7)	41.1 (32.4-50.3)
**Number of patients (*in vivo *drug efficacy studies)^#^**	97	93	128	112	115	104
**Treatment Failure Rate (TFR, %)^#^**	27.8	18.3	16.4	16.1	21.7	11.5

* determined according to *P. falciparum *prevalence and spleen rates (i.e., proportion of individuals with enlarged spleen) of 11-50% in children aged 2-9 years ([29]);

^§ ^([24,26,28]), n, total number of people surveyed; CI, confidence interval; Hb, haemoglobin; Pf, *P. falciparum*; *msp2*, merozoite surface protein 2; nPCR, nested polymerase chain reaction; nd, not determined; ^# ^[30,34]

### Assessment of *in vivo *drug efficacy

Drug efficacy studies were conducted according to the standardized WHO protocol for low to moderate transmission areas [29] and are described in detail elsewhere [30]. Patients were classified according to their clinical and parasitological responses into early treatment failure (ETF), late clinical failure (LCF), late parasitological failure (LPF), or adequate clinical and parasitological response (ACPR) and crude failure rates were PCR-corrected by comparing the *msp2 *(merozoite surface protein 2) genotyping patterns of paired Day 0 and treatment failure samples [31]. Annual treatment failure rates (TFR = ETF + LCF + LPF) of AQ+SP and CQ+SP were pooled for the analysis in the current study.

### Community-based cross-sectional surveys

Cross-sectional surveys were conducted in the catchment areas of each of the health centres using a randomized household approach. To obtain a representative random sample of the parasite population circulating in the corresponding communities (i.e., approximately 100 PCR-positive *P. falciparum *samples from each location), between 300 and 350 blood specimens were collected per community [27]. Overall, a total of 2,013 individuals from randomly selected villages and households were recruited for the community surveys between 2003 and 2005.

Apart from demographic characteristics, collected information included history of sickness (onset, type and duration of symptoms), health facility attendance, purchase or consumption of drugs outside health facilities, and anti-malarial treatment courses received in the preceding year (extracted from health books when available). Axillary temperature was measured with an electronic thermometer and spleen size was assessed in the recumbent position using Hackett's grading system [32]. Blood samples for parasitological examination by microscopy, haemoglobin (Hb) level determination (HemoCue^®^, Ångelholm, Sweden), and molecular identification of parasite genotypes were collected by venepuncture using 2 ml EDTA-Vacutainer™ tubes (BD Biosciences, Allschwil, Switzerland). Plasma was separated by centrifugation and red blood cell pellets were stored frozen until further processing.

### Molecular analyses

DNA was extracted using QIAamp^® ^DNA Blood Kit (Qiagen, Hombrechtikon, Switzerland) according to the manufacturer's instructions. Assessment of SNPs for drug resistant malaria was done for *pfmdr1 *mutations N86Y, Y184F, S1034C, N1042D and D1246Y, *pfcrt *mutations K76T, H97Q, T152A, S163R, A220S, Q271E, N326D/S, I356L/T and R371I, *pfdhfr *mutations A16V, N51I, C59R, S108N/T and I164L, *pfdhps *mutations S436A, A437G, K540E, A581G and A613T/S, and *pfATPase6 *mutations S538R, Q574P, A623E, N683K and S769N. The method is based on parallel PCR amplification of the target sequences followed by primer extension mediated mini-sequencing using fluorochrome-labelled ddNTPs. Subsequent base calling occurs on a microarray upon sequence specific hybridization [33,34]. Multiplicity of infection (MOI) for each sample was assessed by determining the number of *msp2 *genotypes [31].

### Statistical analyses

Data were double entered in EpiData software (version 3.02, Odense, Denmark) and analysis was performed using STATA software (version 10.1; Stata Corp., College Station, Texas). Allele frequencies were calculated separately for each site for the community samples collected in each year, and separately for the health centre samples from both, Karimui and South Wosera collected in 2003. The estimates were very similar to those obtained using a corresponding Bayesian algorithm [35]. Confidence intervals were calculated using bootstrap sampling, in each case based on 10,000 bootstrap samples of the data.

As a measure of genetic differentiation between populations and sample sets , the estimate of the *G*_ST _statistic [36], obtained using the maximum likelihood estimates of the allele frequencies, was used. Overall tests of the null hypothesis that allele frequencies at all four loci result from a sample from a single population were carried out using randomization tests of , in each case using 10,000 random re-assortments of the data to sub-populations.

The frequencies of multi-SNP haplotypes were calculated using the MalHaploFreq programme as described in detail elsewhere [37]. Haplotype frequencies were estimated for each of the main combinations of SNPs possible within each locus. In addition, two-locus haplotype frequencies were estimated for SNPs at the loci involved in SP resistance (i.e., *pfdhfr *and *pfdhps*). Locus-specific comparisons of haplotype frequencies in the different sample sets were carried out using likelihood ratio tests. The likelihood ratio statistic was computed as twice the difference between the log-likelihood of the full model (estimating distinct haplotype frequencies for each sample set) and that of a model estimating common haplotype frequencies for all sample sets included in the comparisons.

### Ethical approval

Scientific approval and ethical clearance for the study was obtained from the Ethikkommission beider Basel (EKBB) and the Medical Research and Advisory Committee (MRAC) of the Ministry of Health in PNG. Informed consent was first requested from all the communities involved and prior to recruitment, individual consent was obtained from each study participant and parents or legal guardians.

## Results

### Characteristics of community survey population

Key characteristics of the sample sets collected from each site between 2003 and 2005 are given in Table 1. Age distribution, sex ratio, and mean axillary temperature were similar between sites, and mean haemoglobin levels were lower in the high transmission areas of the Wosera and the North Coast. Microscopic *P. falciparum *prevalence rates ranged between 13% and 27%, depending on the site and the respective year, and from a total of 2013 community blood samples, 695 (34.5%) were found to be positive for *P. falciparum msp*2 by PCR and prevalence rates determined by a positive *msp2 *PCR ranged from 20% to 41%. MOI determined by *msp2 *PCR was not significantly different between and within sites over time (range: 1.5 to 1.8), but the proportion of multiple versus single infections varied between high and moderate transmission areas. Multiple infections in either *msp*2 or in one or more resistance markers were detected in 436 out of 643 community samples tested, implying the presence of at least 1,247 parasite clones overall.

### *In vivo *drug efficacy

Clinical and parasitological monitoring up to Day 28 between 2003 and 2005 at all three sites was accomplished for a study group of 649 children (Table 1, [30]). In Karimui, the pooled (AQ+SP and CQ+SP) PCR-corrected TFR tended to decrease over the three-year period from 28% to 18% and 16%, respectively, but this was not statistically significant ( = 4.8, *p *= 0.09). In the Wosera, the TFR tended to increase, changing from 16% in 2003 to 22% in 2004, but again this was not statistically significant ( = 1.2, *p *= 0.3). In 2004, the TFR at the North Coast area of Madang was 11.5%.

### Allele and haplotype frequencies in community surveys

Analysis of drug resistance mutations was carried out for 639 (92%) of the community samples. Polymorphisms were found in *pfmdr1 *SNPs N86Y, Y184F and N1042D, *pfcrt *SNPs K76T, A220S, N326D, I356L and S163R, *pfdhfr *SNPs C59R and S108N, and *pfdhps *SNPs A437G and K540E. None of the other SNPs was detected as mutated allele (Table S1, Additional file 1). The proportions of samples found to contain mutant alleles depends on both, the proportion of parasite clones with the mutation (the mutant allele frequency) and the MOIs of the samples [35,37]. The estimated mutant allele frequencies were calculated using the typing results (Table S1, Additional file 1) and the MOIs determined by *msp*2 typing and are given in table S2 (Additional file 2).

High mutant allele frequencies for the CQ relevant markers in *pfcrt *were observed for K76T, N326D and I356L at all three sites (Table S2, Additional file 2). Frequencies of A220S were highest at the North Coast and lower in Karimui and the Wosera. Mutated *pfcrt*S163R alleles were rarely detected and only in Karimui and the Wosera. Frequencies for *pfmdr1*N86Y reached quasi fixed levels in Karimui and at the North Coast, but were substantially lower in the Wosera. *Pfmdr1 *polymorphisms Y184F and N1042D were never detected in samples from Karimui and frequencies were low at the other two sites. Mutant allele frequencies for the SP relevant markers *pfdhfr *S108N and C59R were high at all three sites, but were moderate for *pfdhps*A437G and very low for *pfdhps*K540E.

Haplotype frequencies were estimated for the three SNP combinations *pfmdr1*N86Y+Y184F+N1042D and *pfcrt*K76T+A220S+I356L, and for each of the possible haplotypes of *pfdhfr *plus *pfdhps *arising from the polymorphic SNPs (Table S3, Additional file 3).

### Comparison between sites

The analysis of between-site genetic variation, based on comparing the allele frequencies in all community samples available from each of the three sites, gave a  value of 0.082, which was greater than the value obtained in any of the 10,000 randomizations of the data. This thus corresponds to a P-value < 0.01 suggesting rejection of the null hypothesis that the community samples come from a single parasite population. This  value was considerably higher than the values obtained by comparing between years within sites (Table 2).

**Table 2:** Statistical tests of differences in allele and haplotype frequencies

					Likelihood ratio tests of differences in haplotype frequencies			
Source of variation		Site	Locus	overall	*pfcrt*	*pfmdr1*	*pfdhfr*	*pfdhps*
**Difference between health centre**		Karimui	test statistic	0.004	6.9	0.0	6.3	0.7
**and community samples**			d.f.	n.a.	7	7	3	3
			P-value	0.3	0.4	1.0	0.1	0.9
		South Wosera	test statistic	0.011	30.1	2.3	14.3	14.6
			d.f.	n.a.	7	3	3	3
			P-value	0.02	<0.01	0.5	0.002	0.002
**Difference between years**		Karimui	test statistic	0.035	44.4	2.5	47.8	21.5
**(community samples)**			d.f.	n.a.	14	14	6	6
			P-value	<0.01	<0.01	1.0	<0.01	0.002
		South Wosera	test statistic	0.011	44.8	14.6	16.4	12
			d.f.	n.a.	7	3	3	3
			P-value	0.01	<0.01	0.002	0.001	0.007

d.f., degrees of freedom; n.a., not applicable

**Figure 1:**
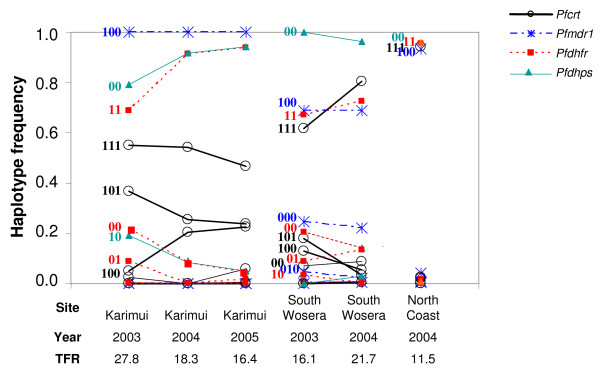
**Single-locus haplotype frequencies in community samples**. TFR, Day 28 treatment failure rate; haplotypes are labelled according to the patterns indicated in Table S3, Additional file 3; labelling is not provided for the superimposed haplotypes with zero or near-zero frequencies.

At the *pfcrt *locus, haplotype frequencies of the wild-type, single K76T or double K76T+I356L mutants were observed with frequencies between 0.02 to 0.36 in Karimui and the Wosera, but were significantly lower in samples from the North Coast, where the haplotype with the triple mutation K76T+A220S+I356L was close to fixation (Table S3, Additional file 3 and Figure 1). At *pfmdr1*, the haplotype comprising the single mutation N86Y predominated at all three sites and was close to fixation in Karimui and the North Coast, but less frequent (0.68) in the Wosera. Fully wild-type *pfdhfr*/*pfdhps *haplotypes were infrequent at all three sites (0.01 to 0.15) and the predominant haplotype at *pfdhfr *was the double mutant S108N+C59R. The *pfdhps*A437G mutation (which always occurred alongside the *pfdhfr *double mutation S108N+C59R) was found in a substantial proportion of samples from the Karimui area, but was much less frequent at the other two sites.

### Comparison of clinical and community samples

**Figure 2:**
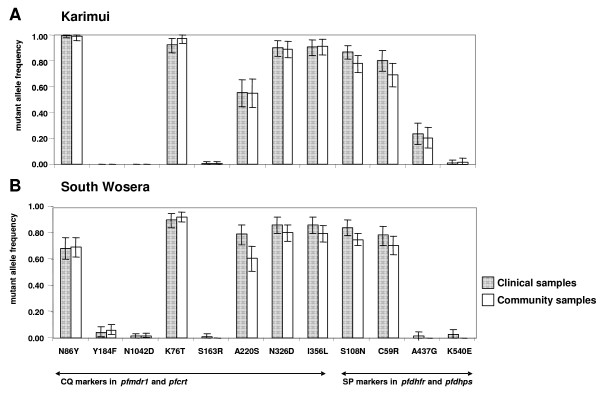
**Comparison of mutant allele frequencies between clinical and community samples at two study sites in Papua New Guinea in the year 2003**. Error bars denote 95% confidence intervals calculated from 10,000 bootstrap samples of the data.

Comparison of the drug resistance profile in community and clinical samples was only possible in the Karimui and South Wosera areas in 2003. In Karimui, the allele frequencies in health centre samples were close to those from community samples for the same year (Table S4, Additional file 4 and Figure 2). In the Wosera, there were small effects of both, year and source of the samples on the allele and haplotype frequencies (both  values 0.011). Despite the rather similar allele frequencies in community and health centre samples, the differences in haplotype frequencies between health centre and community samples were statistically significant for each locus except *pfmdr1 *(Table 2).

### Comparison between years

In Karimui, there was a significant difference between years in the vector of allele frequencies in the community samples, and the value of 0.035 for  measuring inter-annual variation was relatively high for each locus except *pfmdr1*, where a single haplotype appeared to be at fixation. The most obvious changes were related to the allele frequencies of *pfcrt *N326D, I356L and A220S, which all decreased over time, as did *pfdhps*A437G, while the frequencies of the *pfdhfr *mutants increased (Table S2, Additional file 2). In terms of haplotypes, *pfdhfr *and *pfdhps *appeared to be moving towards fixation of the same haplotypes as were already fixed at the North Coast, while there was no indication that any *pfcrt *haplotype was moving towards fixation (Table S3, Additional file 3 and Figure 1).

In the Wosera, the value of  measuring inter-annual variation was lower (0.011), but the variation in haplotype frequencies was statistically significant for all polymorphic loci. While mutations in *pfmdr1 *appeared to be relatively stable, those in the other loci appeared to be moving towards fixation of the same haplotypes as at the North Coast. Triple mutations in *pfcrt *increased in frequency between 2003 and 2004, as did the frequencies of the *pfdhfr*S108N+C59R double and *pfdhps*A437G single mutants (Table S3, Additional file 3 and Figure 1).

### Relationship of allele and haplotype frequencies with treatment failure rates

**Figure 3:**
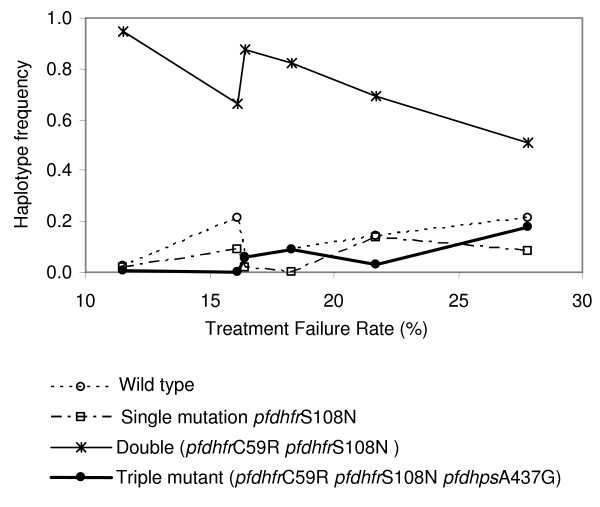
**Two locus (*pfdhfr *plus *pfdhps*) haplotype frequencies in community samples**. Only haplotypes occurring at polymorphic frequencies are shown.

These differences in allele and single-locus haplotype frequencies between sites were not reflected in treatment failure rates (TFR) (Figure 3). In particular, the North Coast site had the lowest TFR, but the highest frequencies of triple mutants in *pfcrt*, of double mutants in *pfdhfr *and of the main polymorphic mutation in *pfmdr1*. Despite the substantial differences in allele and haplotype frequencies between the Wosera and Karimui, the average TFRs at these two sites were similar.

The allelic and single-locus haplotype frequencies in the Wosera seemed to be converging over time with those of the North Coast parasite population (Figure 1), while the TFR in the Wosera was increasing (i.e., diverging from that in the North Coast). At the same time, the general trend in single-locus *pfdhfr *and *pfdhps *mutant haplotype frequencies in Karimui towards the North Coast values accompanied a decrease in TFRs. Thus, the allele frequencies in the Wosera and Karimui could not account for the patterns in TFR. However, a strikingly different pattern was seen with the two-locus haplotypes. While the clinical TFR correlated negatively with the *pfdhfr *double mutant haplotype when this was not linked to the *pfdhps*A437G mutation, the frequency of the *pfdhfr*C59R+S108N/*pfdhps*A437G mutant haplotype increased with the TFR (Figure 3).

## Discussion

The present study was specifically designed to analyse the relationship between parasite genetics in the community and *in vivo *failure rates, but the complexity of this relationship was not initially appreciated. There is substantial evidence linking specific genetic markers to drug resistance *in vitro *and the presence of genetic resistance mutations often predicts treatment failure at the level of the individual patient, but it was observed that these individual level relationships are not reflected in straightforward relationships at the population level between molecular typing results and *in vivo *tests of resistance. A number of possible reasons for this discrepancy were investigated.

One limitation of many studies is that they have included only small numbers of SNPs. The chosen SNPs might prove to be the wrong mutations in the given population or secondary mutations with indirect relationships to resistance might be important. This was addressed by using a DNA microarray-based technology that allowed parallel assessment of many SNPs in several genes [33]. The analysis of a total of 12 polymorphic SNPs, as well as a further 21 SNPs that proved to be uniformly wild-type, enabled to consider all candidate SNPs so far identified. The study populations had highly mutated genes involved in CQR resistance (*pfcrt *and *mdr1*) combined with *pfdhfr *mutations consistent with a moderately pyrimethamine resistant phenotype and the emergence of key mutations in *pfdhps*. The key CQR markers *pfcrt *K76T, N326D and N326D were almost fixed at all three sites, in keeping with other recent findings [38-40]. Similarly, the high allele frequencies for *pfdhfr *S108N and C59R and the resulting high frequency of the double-mutant haplotype are consistent with a recent report from East Sepik Province [41]. Despite this substantial amount of information about each sample, the allele and single-locus haplotype frequencies could still not easily be related to *in vivo *failure rates.

The choice of strategy for sampling the parasite population does not seem to be an important determinant of whether a relationship is seen with treatment failure. The differences between corresponding genetic profiles in health facility and community samples were small in comparison with the differences between sites. In Karimui, the profiles were near identical, and the small differences between the two profiles in the Wosera, which may reflect heterogeneities in access and hence drug pressure for different parts of the catchment area, are too small to influence the choice of sampling strategy. The decision as to whether to sample the community or health centres should therefore be made on logistic grounds. Community sampling can be carried out during a single short visit to a site, while health facility sampling is likely to take longer, but may be more convenient especially if blood sampling for drug resistance can be combined with other procedure(s).

A more important factor in being able to predict treatment failure from parasite genetic data may be the appropriate analysis of the data. Only by analysing two-locus *pfhfr*/*pfdhps *haplotypes coherent relationships between the genetic data and TFRs could be seen, corroborating the previous finding that the *pfdhfr*C59R+S108N/*pfdhps*A437G triple mutant predicts treatment failure best on individual level in PNG [34]. While this haplotype was absent in the North Coast and so cannot account for the treatment failures observed there, increases in haplotype frequency in the other sample sets matched remarkably well the increased TFRs. This is in agreement with studies from Uganda [42] and Laos [43], that also report *pfdhps *mutations to be important markers for unsuccessful treatment response to combination therapy with CQ plus SP, although the *pfdhps *genotype was not indicative for treatment failure with SP monotherapy in Tanzania [44,45] or Ghana [46].

It has been proposed [47-49] that genetic resistance can be measured by the ratio between the prevalence of mutated genotypes to the TFR (the genetic failure index, GFI). A low, reasonably stable GFI would indicate a good marker of resistance. The theoretical properties of GFI are discussed in detail elsewhere [37] where it was pointed out that the GFI for any given SNP could vary widely depending on genotypes at other SNPs modulating the parasites ability to survive therapy. This study quantified this effect, showing that the relationship between SNPs and clinical outcome needs to be based on haplotype frequencies rather than crude prevalence of a single SNP. The estimation of further multi-locus haplotype frequencies may be needed before the full potential of such analysis becomes clear.

Currently, there are technical limitations in the ability to estimate multi-locus haplotype frequencies. Where there are no polyclonal infections, haplotype frequencies can be estimated by simple gene counting. However, 68% of the PCR-positive community samples had multiple *msp2 *types, implying that only 17% of the parasite clones could validly be used to estimate allele frequencies by simple gene counting, making this a very inefficient and imprecise procedure. The estimation of haplotype frequencies from multi-clone samples using the MalHaploFreq programme makes use of substantially more information, but with the current implementation it is not feasible to identify the 3- and 4-locus haplotype frequencies.

Fortunately, there are two ways of avoiding this limitation. Firstly, to date, most resistance appears to have arisen through a few single key SNPs such as *pfcrt*K76T, *pfcrt*A220S and *pfmdr1*N86Y, or by a series of mutations such as the *pfdhfr*108-51-59-164 sequence. Thus, most key multi-locus haplotypes can be characterized at a few numbers of SNPs (e.g. *pfcrt*K76T+*pfmdr1*N86Y, *pfcrt*K76T+*dhfr *I164L, and so on). Secondly, in areas of moderate to high transmission there is likely to be large numbers of mixed infections and hence large amounts of sexual recombination which tends to bring separate genes into linkage disequilibrium (LD). If the genes are in LD then the frequency of multi-gene haplotypes can be obtained simply by multiplying their individual frequencies. Alternately, if mixed infections are rare then haplotypes can be directly observed in single-clone infections and MalHaploFreq is not required.

The inter-site differences in TFR need to be understood in the context of the long-term histories of malaria transmission and drug use at each site, possibly including locality-specific factors, such as clinical practice, genetic background of human population and degree of isolation. After a long history of 4-aminoquinoline use, treatment policy against uncomplicated malaria in PNG changed to combination therapy with AQ or CQ plus SP only in 2000. Both, the TFR and allele/haplotype frequencies, including high frequencies of important markers in *pfdhfr *and *pfdhps*, are therefore the outcomes after only two years of implementation of combination therapy and are probably highly dependent on the baseline frequency of resistant mutations at the time of the policy change.

The sites differed in the frequency of *pfcrt*A220S corresponding to the highly CQ resistant *pfcrt *quadruple mutant haplotype (K76T+N326D+N326D+A220S), which was close to fixation only at the North Coast. Similarly, the *pfmdr1*N86Y mutant was close to fixation in Karimui and at the North Coast (>92%), but was less frequent in the Wosera (71%), and the *pfmdr1 *polymorphisms Y184F and N1042D were absent in Karimui. These genetic differences might simply reflect stochastic differences in source parasite populations, but they could also reflect differences in historical drug pressure. In particular, while *pfcrt *mutations presumably reflect a history of CQ use, SNPs in *pfmdr1 *conferring both, resistance to CQ and altered sensitivity to other drug classes, including quinine (which was used for treatment of severe malaria in PNG), arylaminoalcohols and artemisinin derivates [37,50], are presumably selected by exposure to these. The fixation of a highly CQ resistant haplotype at the North Coast may reflect a history of very high CQ pressure due to good health care provision as well as easy (and unregulated) access to drugs in the nearby town of Madang. In contrast, former 4-aminoquinoline use is assumed to be lower in the remote regions in Karimui and the Wosera, so it is possible that the North Coast pattern is one towards which the others are progressing as a result of drug pressure, yet the North Coast had the lowest TFR. However, former drug pressure with anti-folates in the course of previous mass drug administration campaigns was higher in the two more remote regions [51,52], so a reservoir of anti-folate resistance may have been present there for a long time. Parasite CQR at all three sites may have reached a plateau, while resistance to SP evolved at a different speed depending on both, historical and current anti-folate drug pressure. The frequency of the *pfcrt*A220S mutation increased in the Wosera between 2003 and 2004, most likely as a consequence of inappropriate drug use exerted on a large and heterogeneous parasite population. This arose because of 1) health systems management problems in the area, which lead to an undersupply with SP (local health extension officer at the Kunjingini health centre, personal communication) and 2) drug consumption outside health facilities which was relatively frequent by local standards (10% and 12% of the survey populations in 2003 and 2004, respectively) with AQ or CQ monotherapy distributed by inadequately trained community health workers. These factors may have further enhanced CQR leading to a reduced capacity of CQ to protect SP. Within the same time period, decreasing trends of TFRs were accompanied by decreasing frequencies of *pfcrt*A220S and *pfdhps*A437G in Karimui. Health care provision generally improved due to better medical supply to this remote highland area and the study increased awareness of malaria. The result appears to have been effective delivery of combination therapy to a higher proportion of malaria cases, acting on a relatively small parasite population, therefore preventing the further development and spread of parasite resistance in this moderate transmission area [53,54].

The *in vivo *and molecular data suggest that in the current first-line regimen in PNG, SP, and more specifically sulphadoxine is the effective component. Hence, molecular monitoring of resistance to this component is important under constant treatment policy. However, significant levels of *in vivo *failure are a strong argument for the introduction of an artemisinin-based combination regimen. Once such a regimen is introduced, rapid, easy-to-use and affordable surveillance systems will be needed to monitor emergence and spread of resistance to the components of the new combination. At the same time, surveillance of resistance to withdrawn drugs should also be continued, with the prospect that decreases in resistance may make it possible to reuse one or more of these safe and cheap drugs as partner compounds in combination regimens [55,56].

## Conclusions

In summary, despite there being considerable interest in using molecular data to predict drug failure rates, and hence inform and guide policy choice [5,57], the current analyses showed that the relationship was not as simple as might be hoped. Molecular drug resistance profiles were similar in parasites from community and clinical samples, but these did not closely reflect the longitudinal and geographical variation in *in vivo *drug efficacy observed at health centres. The data confirm that the genetic drug resistance background of the parasite is only one of many factors determining clinical outcome and one which has evolved differently according to epidemiological characteristics as well as history and patterns of drug use in a given area. In particular it was shown that (i) using genetic markers to predict failure rates is much better if based on haplotype frequencies rather than crude prevalence of individual SNPs, and (ii) SNP marker sets will have to be constantly updated and adapted to future monitoring purposes, such as the inclusion of markers for newly introduced or withdrawn drug classes. Finally, this community-based molecular monitoring approach will have to be further evaluated in geographical areas at both extremes of transmission intensity and different drug use patterns (e.g. rural versus urban areas) in order to test its validity as a complementary resistance monitoring tool.

## Abbreviations

*pfmdr1*: *Plasmodium falciparum *multidrug resistance gene 1; *pfcrt*: *Plasmodium falciparum *chloroquine resistance transporter; *pfdhfr*: *Plasmodium falciparum *dihydrofolate reductase; *pfdhps*: *Plasmodium falciparum *dihydropteroate synthase; *pfATPase6*: sarcoendoplasmic reticulum Ca^2+^-ATPase (SERCA) of *P. falciparum*; PCR-RFLP: polymerase chain reaction-restriction fragment length polymorphism.

## Competing interests

The authors declare that they have no competing interests.

## Authors' contributions

JM participated in the coordination of the field and laboratory studies, performed data acquisition and molecular and statistical analyses and drafted the manuscript. TAS, IMH, HPB and BG participated in the design of the study, the statistical analysis and the drafting of the manuscript. IM, AS, OO, MB and JCR participated in the coordination of the field studies. All authors read and approved the final manuscript.

## Supplementary Material

Additional file 1 **Table S1**. Results of molecular typing of community samples. Click here for file

Additional file 2 **Table S2**. Maximum likelihood estimates of mutant allele frequencies from community samples. Click here for file

Additional file 3 **Table S3**. Maximum likelihood estimates of haplotype frequencies from community samples. Click here for file

Additional file 4 **Table S4**. Maximum likelihood estimates of single allele and haplotype frequencies from health centre samples. Click here for file
