# Supplementary material for: Plasmodium falciparum resistance to anti-malarial drugs in Papua New Guinea: evaluation of a community-based approach for the molecular monitoring of resistance
Source: Malar J. 2010 Jan 7;9:8. doi: 10.1186/1475-2875-9-8 (PMC2820042; doi:10.1186/1475-2875-9-8)
Supplement: Additional file 1 — Table S1. Results of molecular typing of community samples. [file 1475-2875-9-8-S1.PDF]

Supplementary table S1: Results of molecular typing of community samples

	Karimui area (Simbu Province)									South Wosera (East Sepik Province)						North Coast (Madang Province)		
**Year**	2003 (N=93)			2004 (N=70)			2005 (N=128)			2003 (N= 106)			2004 (N= 95)			2004 (N=119)		
**SNP**	n_+	n_-	n_{+-}	n_+	n_-	n_{+-}	n_+	n_-	n_{+-}	n_+	n_-	n_{+-}	n_+	n_-	n_{+-}	n_+	n_-	n_{+-}
***pfmdr1* N86Y**	1	93	0	0	70	0	0	128	0	0	105	1	5	86	4	31	76	12
***pfmdr1* Y184F**	93	0	0	70	0	0	128	0	0	106	0	0	91	2	2	114	5	0
***pfmdr1* N1042D**	93	0	0	70	0	0	128	0	0	106	0	0	93	2	0	116	0	3
***pfcrt* K76T**	2	92	0	0	70	0	10	115	3	8	98	0	1	93	1	10	107	2
***pfcrt* S163R**	93	1	0	70	0	0	128	0	0	105	1	0	95	0	0	118	1	0
***pfcrt* A220S**	36	58	0	27	40	3	58	54	16	47	58	1	3	87	5	21	95	3
***pfcrt* N326D**	6	82	6	4	44	22	10	67	51	9	95	2	1	88	6	13	103	3
***pfcrt* I356L**	6	85	3	1	46	23	12	62	54	9	96	1	0	89	6	13	103	3
***pfdhfr* S108N**	9	63	22	6	62	2	3	122	3	7	84	15	3	92	0	13	94	12
***pfdhfr* C59R**	21	63	10	6	62	2	6	118	4	18	84	4	3	89	3	21	90	8
***pfdhps* A437G**	72	16	6	63	7	0	119	6	3	79	21	6	94	1	0	117	2	0
***pfdhps* K540E**	92	2	0	70	0	0	127	1	0	104	1	1	91	3	1	116	3	0

N, number of samples analyzed; SNP, single nucleotide polymorphism; n_+, number of wild-type samples; n_-, number of mutant samples; n_{+-}, samples containing both, wild-type and mutant.
