# Supplementary material for: Plasmodium falciparum resistance to anti-malarial drugs in Papua New Guinea: evaluation of a community-based approach for the molecular monitoring of resistance
Source: Malar J. 2010 Jan 7;9:8. doi: 10.1186/1475-2875-9-8 (PMC2820042; doi:10.1186/1475-2875-9-8)
Supplement: Additional file 2 — Table S2. Maximum likelihood estimates of mutant allele frequencies from community samples. [file 1475-2875-9-8-S2.PDF]

**Supplementary table S2**: Maximum likelihood estimates of mutant allele frequencies from community samples

| | Karimui area (Simbu Province) | | | | | | South Wosera (East Sepik Province) | | | | North Coast (Madang Province) | |
|---|---|---|---|---|---|---|---|---|---|---|---|---|
| **Year** | 2003 | | 2004 | | 2005 | | 2003 | | 2004 | | 2004 | |
| **SNP** | $\hat{p}$ | 95%CI | $\hat{p}$ | 95%CI | $\hat{p}$ | 95%CI | $\hat{p}$ | 95%CI | $\hat{p}$ | 95%CI | $\hat{p}$ | 95%CI |
| ***pfmdr1* N86Y** | 0.99 | (0.96, 1.00) | 1.00 | | 1.00 | | 0.70 | (0.62, 0.77) | 0.70 | (0.61, 0.77) | 0.93 | (0.88, 0.97) |
| ***pfmdr1* Y184F** | 0.00 | | 0.00 | | 0.00 | | 0.06 | (0.03, 0.11) | 0.07 | (0.03, 0.11) | 0.03 | (0.00, 0.05) |
| ***pfmdr1* N1042D** | 0.00 | | 0.00 | | 0.00 | | 0.02 | (0.00, 0.04) | 0.06 | (0.04, 0.10) | 0.01 | (0.00, 0.04) |
| ***pfcrt* K76T** | 0.97 | (0.93, 1.00) | 1.00 | | 0.94 | (0.90, 0.97) | 0.93 | (0.89, 0.97) | 0.90 | (0.85, 0.95) | 0.99 | (0.96, 1.00) |
| ***pfcrt* S163R** | 0.01 | (0.00, 0.02) | 0.00 | | 0.00 | | 0.00 | (0.00, 0.00) | 0.00 | | 0.00 | (0.00, 0.00) |
| ***pfcrt* A220S** | 0.55 | (0.44, 0.66) | 0.54 | (0.42, 0.67) | 0.47 | (0.38, 0.56) | 0.61 | (0.51, 0.70) | 0.82 | (0.75, 0.88) | 0.95 | (0.90, 0.98) |
| ***pfcrt* N326D** | 0.89 | (0.82, 0.95) | 0.77 | (0.69, 0.84) | 0.73 | (0.67, 0.78) | 0.81 | (0.74, 0.87) | 0.85 | (0.79, 0.90) | 0.96 | (0.93, 0.99) |
| ***pfcrt* I356L** | 0.91 | (0.85, 0.97) | 0.80 | (0.73, 0.86) | 0.71 | (0.66, 0.77) | 0.81 | (0.74, 0.87) | 0.85 | (0.80, 0.91) | 0.97 | (0.94, 0.99) |
| ***pfdhfr* S108N** | 0.78 | (0.71, 0.84) | 0.91 | (0.85, 0.97) | 0.95 | (0.90, 0.99) | 0.76 | (0.71, 0.81) | 0.86 | (0.80, 0.91) | 0.97 | (0.93, 1.00) |
| ***pfdhfr* C59R** | 0.69 | (0.60, 0.78) | 0.91 | (0.85, 0.97) | 0.94 | (0.88, 0.98) | 0.71 | (0.64, 0.78) | 0.73 | (0.64, 0.80) | 0.95 | (0.91, 0.99) |
| ***pfdhps* A437G** | 0.20 | (0.13, 0.28) | 0.08 | (0.03, 0.16) | 0.05 | (0.02, 0.10) | 0.00 | (0.00, 0.00) | 0.03 | (0.01, 0.06) | 0.01 | (0.00, 0.03) |
| ***pfdhps* K540E** | 0.02 | (0.00, 0.05) | 0.00 | | 0.01 | (0.00, 0.03) | 0.00 | (0.00, 0.00) | 0.01 | (0.00, 0.02) | 0.03 | (0.00, 0.06) |

SNP, single nucleotide polymorphism; $\hat{p}$ , mutant allele frequency; CI, confidence intervals determined from 10,000 bootstrap samples; nd, not determined. The wild-type allele was fixed for the loci not included in the table.
