# Supplementary material for: Plasmodium falciparum resistance to anti-malarial drugs in Papua New Guinea: evaluation of a community-based approach for the molecular monitoring of resistance
Source: Malar J. 2010 Jan 7;9:8. doi: 10.1186/1475-2875-9-8 (PMC2820042; doi:10.1186/1475-2875-9-8)
Supplement: Additional file 3 — Table S3. Maximum likelihood estimates of haplotype frequencies from community samples. [file 1475-2875-9-8-S3.PDF]

**Supplementary table S3**: Maximum likelihood estimates of haplotype frequencies from community samples

| | Karimui area (Simbu Province) | | | | | | South Wosera (East Sepik Province) | | | | North Coast (Madang Province) | |
|---|---|---|---|---|---|---|---|---|---|---|---|---|
| **Year** | 2003 | | 2004 | | 2005 | | 2003 | | 2004 | | 2004 | |
| **Haplotype** | $\hat{h}$ | 95%CI | $\hat{h}$ | 95%CI | $\hat{h}$ | 95%CI | $\hat{h}$ | 95%CI | $\hat{h}$ | 95%CI | $\hat{h}$ | 95%CI |
| ***pfmdr1* N86Y+Y184F+N1042D** | | | | | | | | | | | | |
| **000** | 0.0 | | 0.0 | | 0.0 | | 0.245 | (0.186, 0.310) | 0.224 | (0.173, 0.281) | 0.034 | (0.012, 0.073) |
| **001** | 0.0 | | 0.0 | | 0.0 | | 0.0 | (0.0, 0.011) | 0.017 | (0.004, 0.040) | 0.0 | (0.0, 0.014) |
| **010** | 0.0 | | 0.0 | | 0.0 | | 0.045 | (0.021, 0.079) | 0.023 | (0.008, 0.049) | 0.013 | (0.002, 0.041) |
| **011** | 0.0 | | 0.0 | | 0.0 | | 0.009 | (0.001, 0.028) | 0.045 | (0.022, 0.077) | 0.013 | (0.002, 0.041) |
| **100** | 1.0 | | 1.0 | | 1.0 | | 0.686 | (0.617, 0.750) | 0.681 | (0.619, 0.738) | 0.940 | (0.893, 0.972) |
| **101** | 0.0 | | 0.0 | | 0.0 | | 0.009 | (0.001, 0.028) | 0.010 | (0.001, 0.032) | 0.0 | (0.0, 0.014) |
| **110** | 0.0 | | 0.0 | | 0.0 | | 0.006 | (0.0, 0.029) | 0.0 | (0.0, 0.008) | 0.0 | (0.0, 0.016) |
| **111** | 0.0 | | 0.0 | | 0.0 | | 0.0 | (0.0, 0.010) | 0.0 | (0.0, 0.009) | 0.0 | (0.0, 0.014) |
| ***pfcrt* K76T+A220S+I356L** | | | | | | | | | | | | |
| **000** | 0.026 | (0.007, 0.060) | 0.0 | (0.0, 0.016) | 0.059 | (0.033, 0.094) | 0.069 | (0.040, 0.108) | 0.088 | (0.057, 0.127) | 0.010 | (0.0, 0.031) |
| **001** | 0.0 | (0.0, 0.014) | 0.0 | (0.0, 0.016) | 0.005 | (0.0, 0.020) | 0.0 | (0.0, 0.010) | 0.004 | (0.0, 0.018) | 0.0 | (0.0, 0.031) |
| **010** | 0.0 | (0.0, 0.014) | 0.0 | (0.0, 0.016) | 0.0 | (0.0, 0.009) | 0.0 | (0.0, 0.009) | 0.004 | (0.0, 0.017) | 0.0 | (0.0, 0.022) |
| **011** | 0.0 | (0.0, 0.014) | 0.0 | (0.0, 0.016) | 0.0 | (0.0, 0.009) | 0.0 | (0.0, 0.009) | 0.004 | (0.0, 0.017) | 0.005 | (0.0, 0.022) |
| **100** | 0.052 | (0.023, 0.095) | 0.204 | (0.138, 0.2820) | 0.227 | (0.176, 0.284) | 0.130 | (0.088, 0.182) | 0.054 | (0.028, 0.089) | 0.020 | (0.006, 0.053) |
| **101** | 0.368 | (0.292, 0.447) | 0.255 | (0.182, 0.338) | 0.238 | (0.185, 0.297) | 0.181 | (0.130, 0.240) | 0.034 | (0.015, 0.068) | 0.025 | (0.009, 0.055) |
| **110** | 0.006 | (0.0, 0.029) | 0.0 | (0.0, 0.017) | 0.005 | (0.0, 0.021) | 0.005 | (0.0, 0.021) | 0.008 | (0.001, 0.027) | 0.0 | (0.0, 0.011) |
| **111** | 0.548 | (0.468, 0.628) | 0.542 | (0.454, 0.629) | 0.467 | (0.403, 0.531) | 0.615 | (0.548, 0.679) | 0.803 | (0.750, 0.849) | 0.940 | (0.901, 0.968) |
| ***pfdhfr* C59R+S108N** | | | | | | | | | | | | |
| **00** | 0.227 | (0.162, 0.301) | 0.086 | (0.044, 0.144) | 0.048 | (0.025, 0.080) | 0.210 | (0.155, 0.273) | 0.140 | (0.101, 0.186) | 0.010 | (0.011, 0.060) |
| **01** | 0.086 | (0.046, 0.141) | 0.0 | (0.0, 0.016) | 0.016 | (0.005, 0.038) | 0.086 | (0.050, 0.135) | 0.133 | (0.094, 0.178) | 0.015 | (0.003, 0.040) |
| **10** | 0.0 | (0.0, 0.015) | 0.0 | (0.0, 0.017) | 0.0 | (0.0, 0.009) | 0.032 | (0.011, 0.069) | 0.0 | (0.0, 0.008) | 0.0 | (0.0, 0.011) |
| **11** | 0.687 | (0.608, 0.761) | 0.914 | (0.855, 0.956) | 0.936 | (0.901, 0.963) | 0.671 | (0.601, 0.737) | 0.727 | (0.670, 0.780) | 0.955 | (0.920, 0.979) |
| ***pfdhps* A437G+K540E** | | | | | | | | | | | | |
| **00** | 0.790 | (0.719, 0.851) | 0.915 | (0.857, 0.956) | 0.941 | (0.906, 0.967) | 1.0 | | 0.963 | (0.935, 0.982) | 0.960 | (0.927, 0.982) |
| **01** | 0.006 | (0.0, 0.029) | 0.0 | (0.0, 0.016) | 0.008 | (0.001, 0.025) | 0.0 | | 0.007 | (0.001, 0.024) | 0.030 | (0.011, 0.060) |
| **10** | 0.191 | (0.132, 0.260) | 0.085 | (0.044, 0.143) | 0.051 | (0.028, 0.085) | 0.0 | | 0.029 | (0.013, 0.055) | 0.010 | (0.001, 0.031) |
| **11** | 0.013 | (0.002, 0.041) | 0.0 | (0.0, 0.016) | 0.0 | (0.0, 0.009) | 0.0 | | 0.0 | (0.0, 0.008) | 0.0 | (0.0, 0.011) |
| ***pfdhfr* C59R+S108N+*pfdhps*A437G** | | | | | | | | | | | | |
| **000** | 0.214 | (0.149, 0.288) | 0.091 | (0.045, 0.156) | 0.053 | (0.028, 0.087) | 0.215 | (0.159, 0.279) | 0.143 | (0.103, 0.189) | 0.026 | (0.007, 0.060) |
| **001** | 0.016 | (0.002, 0.049) | 0.0 | (0.0, 0.019) | 0.0 | (0.0, 0.009) | 0.0 | (0.0, 0.009) | 0.0 | (0.0, 0.011) | 0.0 | (0.0, 0.014) |
| **010** | 0.083 | (0.043, 0.138) | 0.0 | (0.0, 0.019) | 0.018 | (0.005, 0.042) | 0.089 | (0.051, 0.139) | 0.135 | (0.096, 0.181) | 0.020 | (0.004, 0.051) |
| **011** | 0.007 | (0.0, 0.032) | 0.0 | (0.0, 0.019) | 0.0 | (0.0, 0.009) | 0.0 | (0.0, 0.009) | 0.0 | (0.0, 0.009) | 0.0 | (0.0, 0.014) |
| **100** | 0.0 | (0.0, 0.016) | 0.0 | (0.0, 0.019) | 0.0 | (0.0, 0.010) | 0.033 | (0.011, 0.071) | 0.0 | (0.0, 0.008) | 0.0 | (0.0, 0.014) |
| **101** | 0.0 | (0.0, 0.016) | 0.0 | (0.0, 0.019) | 0.0 | (0.0, 0.009) | 0.0 | (0.0, 0.009) | 0.0 | (0.0, 0.010) | 0.0 | (0.0, 0.014) |
| **110** | 0.506 | (0.422, 0.590) | 0.820 | (0.740, 0.885) | 0.873 | (0.825, 0.913) | 0.663 | (0.591, 0.729) | 0.693 | (0.634, 0.746) | 0.948 | (0.903, 0.976) |
| **111** | 0.175 | (0.117, 0.244) | 0.089 | (0.045, 0.152) | 0.057 | (0.031, 0.092) | 0.0 | (0.0, 0.009) | 0.030 | (0.013, 0.056) | 0.006 | (0.0, 0.030) |

$\hat{h}$, estimated haplotype frequency; CI, confidence intervals; 0, wild-type allele; 1, mutated allele.
