# Supplementary material for: Plasmodium falciparum resistance to anti-malarial drugs in Papua New Guinea: evaluation of a community-based approach for the molecular monitoring of resistance
Source: Malar J. 2010 Jan 7;9:8. doi: 10.1186/1475-2875-9-8 (PMC2820042; doi:10.1186/1475-2875-9-8)
Supplement: Additional file 4 — Table S4. Maximum likelihood estimates of single allele and haplotype frequencies from health centre samples. [file 1475-2875-9-8-S4.PDF]

Supplementary table S4: Maximum likelihood estimates of single allele and haplotype frequencies from health centre samples

	**Sigimaru HC (Karimui)** (N=97)			**Kunjingini HC (South Wosera)** (N=112)		
**SNP**	$\hat{p}$	95%CI		$\hat{p}$	95%CI	
***pfmdr1* N86Y**	0.69	(0.60,	0.77)	0.99	(0.98,	1.00)
***pfmdr1* Y184F**	0.05	(0.01,	0.09)	0.00		
***pfmdr1* N1042D**	0.01	(0.00,	0.03)	0.00		
***pfcrt* K76T**	0.91	(0.85,	0.96)	0.92	(0.86,	0.97)
***pfcrt* S163R**	0.01	(0.00,	0.03)	0.01	(0.00,	0.02)
***pfcrt* A220S**	0.80	(0.72,	0.87)	0.55	(0.45,	0.66)
***pfcrt* N326D**	0.87	(0.80,	0.93)	0.90	(0.84,	0.96)
***pfcrt* I356L**	0.87	(0.80,	0.93)	0.91	(0.84,	0.96)
***pfdhfr* S108N**	0.85	(0.79,	0.90)	0.87	(0.81,	0.92)
***pfdhfr* C59R**	0.79	(0.71,	0.86)	0.80	(0.72,	0.88)
***pfdhps* A437G**	0.02	(0.00,	0.05)	0.23	(0.15,	0.32)
***pfdhps* K540E**	0.03	(0.00,	0.07)	0.01	(0.00,	0.03)
**Haplotype**						
***pfmdr1* N86Y+Y184F+N1042D**	$\hat{h}$	95%CI		$\hat{h}$	95%CI	
**000**	0.006	(0.0,	0.026)	0.261	(0.203,	0.325)
**001**	0.0	(0.0,	0.012)	0.0	(0.0,	0.010)
**010**	0.994	(0.974,	0.999)	0.687	(0.620,	0.749)
**011**	0.0	(0.0,	0.011)	0.005	(0.0,	0.021)
**100**	0.0	(0.0,	0.012)	0.038	(0.017,	0.071)
**101**	0.0	(0.0,	0.012)	0.010	(0.001,	0.030)
**110**	0.0	(0.0,	0.011)	0.0	(0.0,	0.010)
**111**	0.0	(0.0	0.012)	0.0	(0.0,	0.010)
***pfcrt* K76T+A220S+I356L**						
**000**	0.072	(0.038,	0.119)	0.090	(0.055,	0.135)
**001**	0.0	(0.0,	0.013)	0.0	(0.0,	0.010)
**010**	0.0	(0.0,	0.013)	0.0	(0.0,	0.010)
**011**	0.0	(0.0,	0.013)	0.0	(0.0,	0.010)
**100**	0.019	(0.004,	0.048)	0.030	(0.011,	0.061)
**101**	0.343	(0.272,	0.418)	0.082	(0.049,	0.126)
**110**	0.0	(0.0,	0.013)	0.005	(0.0,	0.022)
**111**	0.566	(0.488,	0.642)	0.793	(0.732,	0.845)
***pfdhfr* C59R+S108N**						
**00**	0.135	(0.089,	0.191)	0.152	(0.106,	0.206)
**01**	0.062	(0.032,	0.105)	0.056	(0.029,	0.095)
**10**	0.0	(0.0,	0.012)	0.0	(0.0,	0.011)
**11**	0.803	(0.740,	0.859)	0.792	(0.733,	0.845)
***pfdhps* A437G+K540E**						
**00**	0.753	(0.684,	0.815)	0.951	(0.915,	0.976)
**01**	0.006	(0.0,	0.026)	0.029	(0.011,	0.059)
**10**	0.235	(0.175,	0.304)	0.020	(0.005,	0.045)
**11**	0.006	(0.0,	0.026)	0.0	(0.0,	0.010)

N, number of samples analzsed; SNP, single nucleotide polymorphism; $\hat{p}$, mutant allele frequency; $\hat{h}$, estimated haplotype frequency; 0 wild-type allele; 1, mutated allele; CI, confidence intervals determined from 10,000 bootstrap samples.
